# World Health Organization International Standard to Harmonize Assays for Detection of Hepatitis E Virus RNA

**DOI:** 10.3201/eid1905.121845

**Published:** 2013-05

**Authors:** Sally A. Baylis, Johannes Blümel, Saeko Mizusawa, Keiji Matsubayashi, Hidekatsu Sakata, Yoshiaki Okada, C. Micha Nübling, Kay-Martin O. Hanschmann, the HEV Collaborative Study Group

**Affiliations:** Paul-Ehrlich-Institut, Langen, Germany (S.A. Baylis, J. Blümel, C.M. Nübling, K.-M.O. Hanschmann);; National Institute of Infectious Diseases, Tokyo, Japan (S. Mizusawa, Y. Okada);; Japanese Red Cross Hokkaido Block Blood Center, Sapporo, Japan (K. Matsubayashi, H. Sakata)

**Keywords:** hepatitis E virus, HEV, NAT, standardization, RNA, hepatitis, nucleic acid amplification technique, detection, international standard, WHO, World Health Organization, assays, genotype, subgenotype, genotype 3, genotype 3a, strains, viruses

## Abstract

Nucleic acid amplification technique–based assays are a primary method for the detection of acute hepatitis E virus (HEV) infection, but assay sensitivity can vary widely. To improve interlaboratory results for the detection and quantification of HEV RNA, a candidate World Health Organization (WHO) International Standard (IS) strain was evaluated in a collaborative study involving 23 laboratories from 10 countries. The IS, code number 6329/10, was formulated by using a genotype 3a HEV strain from a blood donation, diluted in pooled human plasma and lyophilized. A Japanese national standard, representing a genotype 3b HEV strain, was prepared and evaluated in parallel. The potencies of the standards were determined by qualitative and quantitative assays. Assay variability was substantially reduced when HEV RNA concentrations were expressed relative to the IS. Thus, WHO has established 6329/10 as the IS for HEV RNA, with a unitage of 250,000 International Units per milliliter.

Hepatitis E virus (HEV) is a nonenveloped, single-stranded RNA virus belonging to the family *Hepeviridae* (*1*,*2*). In developing countries, HEV is a major cause of acute hepatitis, transmitted by the fecal–oral route and associated with contamination of drinking water. In industrialized countries, reports of HEV infection have been uncommon but are being reported more frequently; some cases are imported after travel to HEV-endemic areas, but reports of autochthonous cases are also increasing, and infection with HEV appears to be more prevalent than originally believed (*3*). Prospects for control of HEV infection are encouraged by recent efforts in vaccine development (*4*,*5*). 

Four main genotypes of HEV, representing a single serotype, infect humans. Genotype 1 viruses are found mainly in Africa and Asia and genotype 2 in Africa and Central America; it is in these areas that prevention of HEV infection by vaccination would be most beneficial. Genotypes 3 and 4 viruses are generally less pathogenic, although some exceptions have been reported, particularly for genotype 4; these genotypes infect not only humans but also animals such as swine, wild boar, and deer. Although genotype 4 strains have mainly been restricted to parts of Asia, genotype 3 viruses are found widely throughout the world. Zoonotic transmission of HEV genotypes 3 and 4 to humans can occur by consumption of contaminated meat or meat products or by contact with infected animals (*6*,*7*). Shellfish, such as bivalve mollusks, have also been shown to act as reservoirs for HEV (*8*).

An alternate route of transmission of HEV by transfusion of blood components has been reported in Japan (*9**,**10*), the United Kingdom (*11*), and France (*12*,*13*). Studies in Japan (*14*) and the People’s Republic of China (*15*) have identified acute HEV infections in blood donors, confirmed by the detection of HEV RNA. Analysis of blood and plasma donors in Europe has identified HEV-infected donors in Germany (*16*–*20*), Sweden (*18*), and England (*21*). Transmission of HEV by solid organ transplantation has also been reported (*22*). Rates of HEV infection may be underreported in some countries, and misdiagnosis of HEV infection also occurs. For example, in some cases of suspected drug-induced liver injury, HEV has been determined as the cause (*23*). In one such recent case, HEV was shown to have been transmitted by blood transfusion (*13*).

Infection with HEV may cause particularly severe illness in pregnant women and in persons who have preexisting liver disease. Chronic infection with HEV genotype 3 is an emerging problem among solid organ transplant recipients and may also occur in persons with HIV and certain hematologic disorders (*24*). In patients with chronic infection, viral loads are monitored to investigate the efficacy of antiviral treatment (*25*,*26*) and effects of reduction of immunosuppressive therapy (*27*). 

HEV infection is diagnosed on the basis of detection of specific antibodies (IgM and IgG), but the sensitivity and specificity of these assays is not optimal (*28*–*30*). Analysis of HEV RNA by using nucleic acid amplification techniques (NATs) is also used for diagnosis; this method can identify active infection and help confirm serologic results (*31*). Several NAT assays have been reported for the detection of HEV RNA in serum and plasma or fecal samples: conventional reverse transcription PCR (RT-PCR) and nested protocols (*32*), real-time RT-PCR, and reverse transcription loop-mediated isothermal amplification (*33*). The NATs include generic assays designed for the detection of HEV genotypes 1–4 (*34*,*35*). 

In 2009, the World Health Organization (WHO) Expert Committee on Biological Standardization endorsed a proposal by the Paul-Ehrlich-Institut (PEI) to prepare an International Standard (IS) for HEV RNA for use in NAT-based assays. PEI recently completed an initial study that investigated the performance of HEV NAT assays in detection of HEV infection (*36*). In that study, dilution panels of HEV genotype 3 and 4 strains underwent blinded testing in laboratories that had experience in detection of HEV RNA. Results demonstrated wide variations in assay sensitivity (in the order of 100- to 1,000-fold for most assays). 

After the initial study, 2 virus strains included in the panel (*36*) were selected for further development of a candidate IS for the WHO, and a candidate Japanese national standard (done in collaboration with the National Institute of Infectious Diseases in Tokyo). These viruses belong to genotype 3, which is widely distributed, and were genotype 3a and 3b strains, which were equally well detected in the initial study. The strains were derived from plasma samples that had sufficient titers of HEV RNA to prepare standards of good potency. An international collaborative study was conducted to establish the respective standards, demonstrate suitability for use, evaluate potency, and assign an internationally agreed-upon unitage.

## Methods

### Preparation of Materials

The 2 HEV strains selected for the preparation of the candidate WHO IS and candidate Japanese national standard were genotype 3a strain HRC-HE104 and genotype 3b strain JRC-HE3, respectively. The HEV-positive plasma donations were kindly provided by the Japanese Red Cross Society Blood Service Headquarters (Tokyo, Japan). Characterization of the stock virus strains is shown in Table 1. 

**Table 1:** HEV strains diluted and lyophilized as candidate standards in study to establish a WHO International Standard for HEV RNA NAT-based assays*

Virus strain	HEV RNA, copies/mL	Genotype	GenBank accession no.	IgM/IgG against HEV	Alanine aminotransferase, IU/L
HRC-HE104	1.6 × 10^7^	3a	AB630970	−/−	36
JRC-HE3	2.5 × 10^7^	3b	AB630971	+/−	398

*Strains were provided by the Japanese Red Cross Society Blood Service Headquarters, Tokyo, Japan. HEV, hepatitis E virus; WHO, World Health Organization; NAT, nucleic acid amplification technique.

The samples were tested for IgG/IgM against HEV by using an HEV enzyme immunoassay (Institute of Immunology Co., Ltd., Tokyo, Japan). Full-length sequences of the HEV strains were determined as described (*37*). Phylogenetic analyses were conducted by using MEGA version 5.05 (*38*), and HEV genotype and subgenotype were determined as described (*39*). The nucleotide sequences of HRC-HE104 and JRC-HE3 were deposited into GenBank under accession nos. AB630970 and AB630971, respectively.

The target HEV RNA concentration for the 2 bulk standard preparations was ≈5.5 log_10_ HEV RNA copies/mL, on the basis of the concentrations determined in the initial study (*36*). The 2 virus strains were negative when tested for hepatitis B virus, hepatitis C virus, and HIV-1/2 by using the Cobas TaqScreen MPX test (Roche Molecular Systems Inc., Branchburg, NJ, USA). The samples were diluted by using pooled citrated plasma (*36*) that had tested negative by NAT for hepatitis B virus, hepatitis C virus, and HIV-1/2, and HEV and was also negative for antibodies against HEV by using the recomWell IgG and IgM enzyme immunoassays (Mikrogen GmbH, Neuried, Germany). The diluted plasma was placed into 4-mL screw-cap glass vials, freeze dried, filled with nitrogen, sealed with rubber stoppers, and stored at −20°C. Stability studies demonstrated no substantial change in HEV RNA concentration after freeze drying or after 10 months of storage at −20°C (the usual temperature), +4°C, and +20 to +26°C, compared with samples stored at <−80°C.

### Study Design

The collaborative study was conducted by 24 laboratories from 10 countries; each laboratory was randomly assigned a code number. The samples analyzed in the study were coded sample 1 and sample 2 (replicates of the candidate WHO IS) and sample 3 and sample 4 (replicates of the candidate Japanese national standard). Samples were shipped to participants at ambient temperature. Participants tested the samples by using the laboratory’s routine assays for HEV RNA, in 4 separate assay runs, using fresh vials of each sample for each run. Quantitative assay results falling within the linear range of the assays were reported in copies/mL. For qualitative assays, participants assayed each sample by a series of 1.0-log_10_ dilution steps to obtain an initial estimate of an endpoint and then, in 3 subsequent runs, assayed 0.5-log_10_ dilutions around the endpoint determined in the first run.

### Statistical Methods

#### Quantitative Assays

Evaluation of quantitative assays was restricted to dilutions of 0.0 log_10_ to −2.5 log_10_, a range over which the assays of most participants produced comparable data. For comparison of laboratories, the replicate results of each laboratory, corrected for the dilution factor, were combined as the arithmetic mean of log_10_ copies/mL. Furthermore, these estimates were combined to obtain an overall estimation for each sample by means of a mixed linear model, using laboratory and log_10_ dilution as random factors.

#### Qualitative Assays

The data from all assays were pooled to give a series of values for number positive/number tested at each dilution. For each participant, these pooled results were evaluated by means of probit analysis to estimate the concentration at which 50% of the samples tested were positive; for assays in which the change from complete negative to complete positive results occurred in <2 dilution steps, the Spearman-Kaerber method was applied for estimation. The calculated endpoint was used to give estimates expressed in log_10_ NAT-detectable units/mL, after correcting for the equivalent volume of the test sample.

### Relative Potencies

For quantitative assays, potencies of samples 2, 3, and 4 were estimated relative to sample 1 by using parallel-line analysis of log-transformed data. For qualitative assays, relative potencies were determined by using parallel-line analysis of probit-transformed data. Statistical analyses were performed by using SAS/STAT version 9.3 (SAS Institute, Cary, NC, USA). Estimation of endpoint dilution and relative potencies was performed by using CombiStats version 4.0 (European Directorate for the Quality of Medicines and HealthCare/Council of Europe, Strasbourg, France).

## Results

Data were returned by 23 of the 24 participating laboratories; 20 sets of qualitative data and 14 sets of quantitative data were evaluated. The assays used by the participants are shown in Technical Appendix Table 1. All assays were developed in-house and were either conventional or nested RT-PCRs or based on real-time RT-PCR.

### Quantitative and Qualitative Assay Results

**Figure 1:**
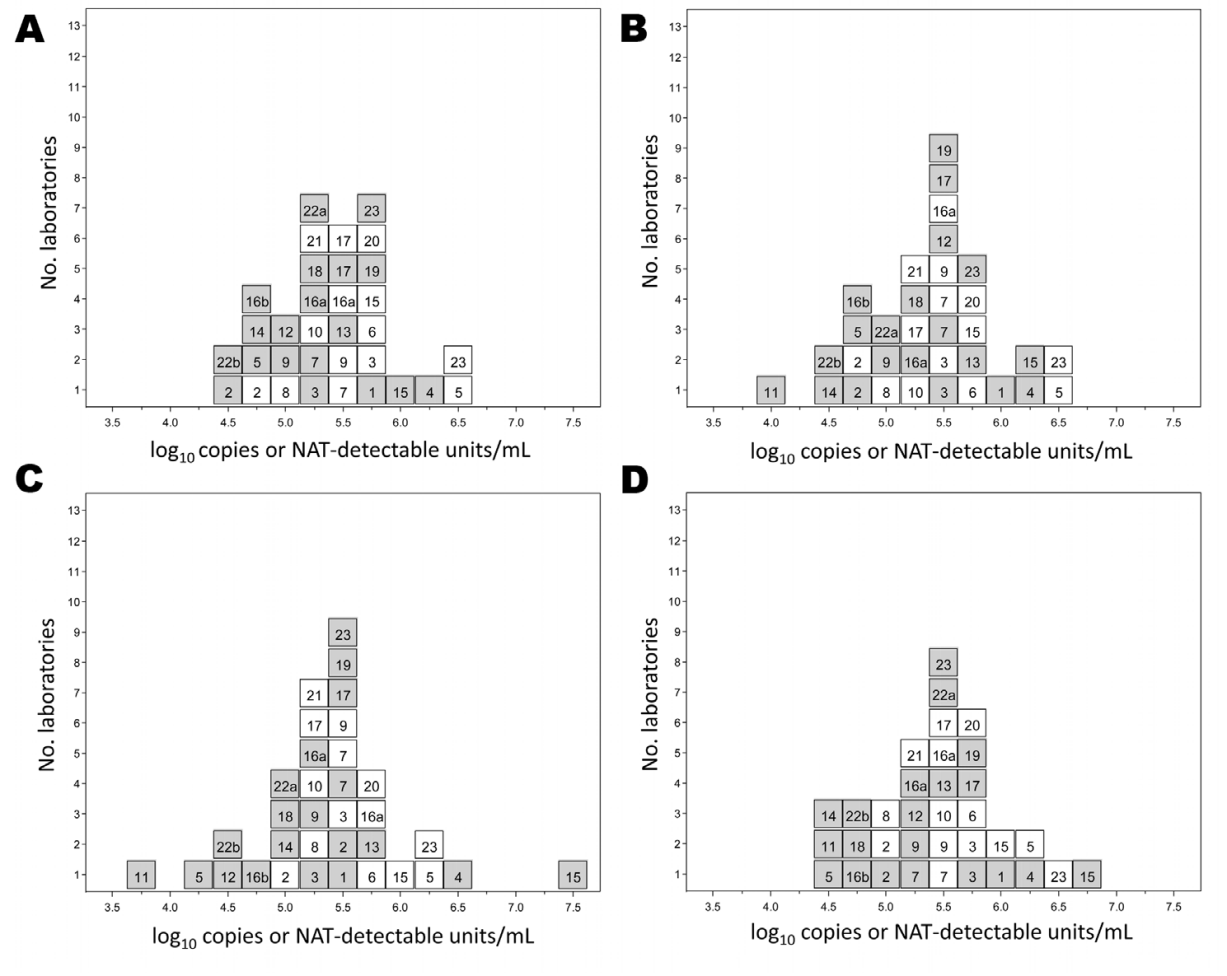
Histograms showing results for quantitative and qualitative assays conducted by 23 laboratories for the determination of the hepatitis E virus (HEV) RNA content of sample 1 (A), sample 2 (B), sample 3 (C), and sample 4 (D). White indicates quantitative assays (log_10_ copies/mL); gray indicates qualitative assays (log_10_ nucleic acid amplification technique (NAT)–detectable units/mL). Number of laboratories is indicated on the vertical axis. Laboratory code numbers are indicated in the respective boxes.

**Figure 2:**
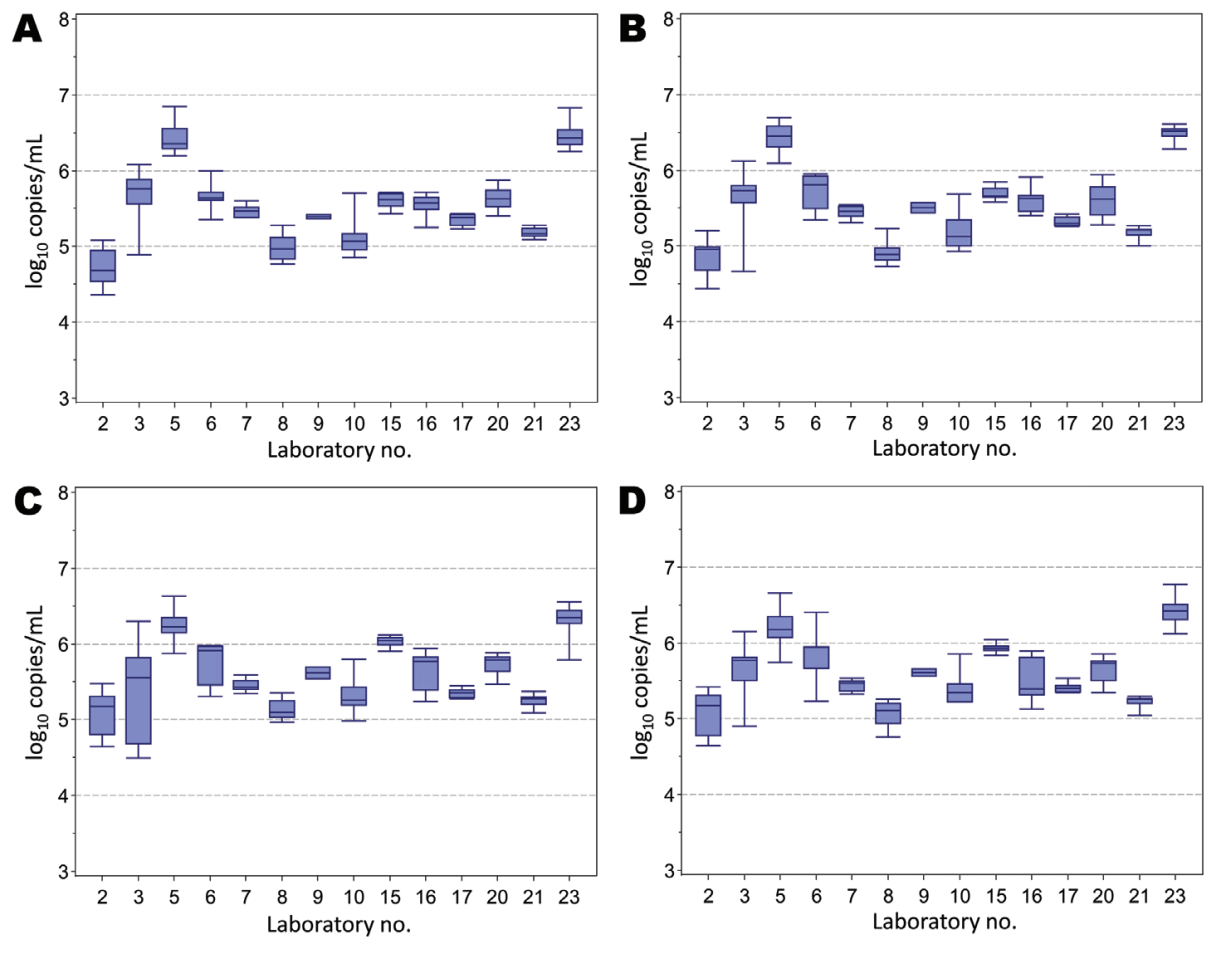
Box and whisker plots of the results for quantitative assays (log_10_ copies/mL) conducted by laboratories for the determination of the hepatitis E virus (HEV) RNA content of sample 1 (A), sample 2 (B), sample 3 (C), and sample 4 (D). Box indicates interquartile range; line within box indicates median; whiskers indicate minimum and maximum values observed. Laboratory code numbers are given on the horizontal axis.

Laboratory mean estimates for quantitative assays (in log10 copies/mL) and qualitative assays (in NAT-detectable log10 units/mL) for the HEV preparations are shown in histogram form in Figure 1, which shows that laboratory means are more variable for the qualitative assays than the quantitative assays, reflecting different assay sensitivities and lack of standardization. The individual laboratory means are given in Technical Appendix Tables 2 and 3; relative variation of the individual laboratory estimates for the quantitative assays is illustrated by the box-and-whisker plots in Figure 2. Intralaboratory variation was lower than the interlaboratory variation for both types of assays (data not shown).

**Table 2:** Overall mean estimates from quantitative and qualitative assays of HEV samples in study to establish a WHO International Standard for HEV RNA NAT-based assays*

Assay type and sample	No.	Mean (95% CI)†	SD	% CV
Quantitative				
1	123	5.58 (5.32–5.85)	0.54	98
2	125	5.60 (5.33–5.87)	0.53	94
1 + 2	248	5.59 (5.33–5.86)	0.55	99
3	124	5.66 (5.40–5.93)	0.45	77
4	125	5.66 (5.40–5.93)	0.44	76
3 + 4	249	5.66 (5.40–5.93)	0.44	76
Qualitative				
1	19	5.25 (5.01–5.50)	0.51	150
2	20	5.26 (4.97–5.56)	0.62	179
1 + 2	39	5.26 (5.08–5.44)	0.56	163
3	20	5.27 (4.90–5.64)	0.79	226
4	20	5.31 (5.02–5.61)	0.64	183
3 + 4	40	5.29 (5.07–5.52)	0.71	202

*Samples 1 and 2, replicate samples of the candidate WHO International Standard; samples 3 and 4, replicate samples of the candidate Japanese national standard. HEV, hepatitis E virus; WHO, World Health Organization; NAT, nucleic acid amplification technique; no., no. dilutions analyzed (in linear range for quantitative assays); % CV, geometric coefficient of variation. †Values are log_10_ copies/mL for quantitative and log_10_ NAT–detectable units/mL for qualitative assays.

**Table 3:** Overall mean potencies of samples 2, 3, and 4 relative to sample 1 from quantitative and qualitative analysis of HEV samples in study to establish a WHO International Standard for HEV RNA NAT-based assays*

Sample and assay type	No.	Mean (95% CI)†	SD	% CV
Sample 2				
Quantitative	19	5.46 (5.35–5.58)	0.23	3
Qualitative	13	5.42 (5.38–5.46)	0.07	1
Combined	32	5.45 (5.38–5.51)	0.18	2
Sample 3				
Quantitative	20	5.45 (5.27–5.65)	0.43	5
Qualitative	13	5.48 (5.37–5.59)	0.18	2
Combined	33	5.46 (5.35–5.58)	0.35	4
Sample 4				
Quantitative	20	5.51 (5.38–5.64)	0.29	3
Qualitative	13	5.47 (5.36–5.59)	0.19	2
Combined	33	5.49 (5.41–5.58)	0.25	3

*Mean potency values were determined by assigning a value of 5.39 log_10_ units/mL for sample 1. Samples 1 and 2, replicate samples of the candidate WHO International Standard; samples 3 and 4, replicate samples of the candidate Japanese national standard. HEV, hepatitis E virus; WHO, World Health Organization; NAT, nucleic acid amplification technique; no., no. dilutions analyzed (in linear range for quantitative assays); % CV, geometric coefficient of variation. †Values are log_10_ copies/mL for quantitative and log_10_ NAT technique–detectable units/mL for qualitative assays.

### Determination of Overall Laboratory Means

The means for all the laboratories performing quantitative assays are shown in Table 2. The means for sample 1 and sample 2, replicates for the candidate WHO IS, were 5.58 log_10_ and 5.60 log_10_ copies/mL HEV RNA, respectively, with good agreement between the replicate samples. The candidate Japanese national standard showed identical mean results of 5.66 log_10_ copies/mL HEV RNA for replicate samples 3 and 4.

The means for all the laboratories performing qualitative assays are also shown in Table 2; again, there was good agreement between the duplicate samples. Results for the qualitative assays showed 0.3-log_10_ lower mean estimates and a higher SD than those for the quantitative assays. The combined mean values for the replicate samples for both types of assays are shown in Table 2.

### Relative Potencies

On the basis of the combined data from both qualitative and quantitative assays, the candidate WHO standard was determined to have a potency of 5.39 log_10_ units/mL (95% CI 5.15–5.63). This value was calculated with a combined endpoint evaluation of qualitative and quantitative data (restricted to dilutions in the range of 0.0 log_10_ to −2.5 log_10_) by means of a mixed linear model.

**Figure 3:**
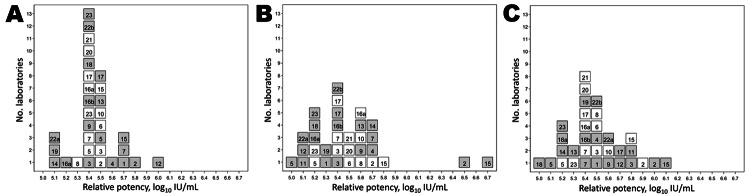
Histograms showing potencies of sample 2 (A), sample 3 (B), and sample 4 (C) compared with sample 1, the candidate World Health Organization International Standard for hepatitis E virus RNA for nucleic acid amplification technique (NAT)–based assays. White indicates quantitative assays (log_10_ copies/mL); gray indicates qualitative assays (log_10_ NAT–detectable units/mL). Number of laboratories is indicated on the vertical axis. Laboratory code numbers are indicated in the respective boxes.

The potencies of samples 2, 3, and 4 were calculated relative to sample 1, taking the value of sample 1 as 5.39 log_10_ units/mL. The relative potencies for the quantitative and qualitative assays are shown in Technical Appendix Tables 4 and 5, respectively. Table 3 summarizes the overall mean potencies relative to sample 1, with the 95% CIs, SDs, and geometric coefficients of variation. For the quantitative data from laboratory 9, no potency could be estimated by endpoint evaluation because only 1 dilution was tested for each sample. The data are plotted in histogram form in Figure 3. 

The data demonstrate that expressing the results as potencies relative to sample 1 (set as a standard with an assumed unitage of 5.39 log_10_ units/mL) results in a marked improvement in the agreement between the majority of methods and laboratories, as evidenced by the reduction in SDs. Furthermore, these data provide some evidence for commutability of the candidate standard for evaluation of HEV from infected persons, because samples 1 and 2 represent a different strain of HEV compared with samples 3 and 4.

## Discussion

In this study, a wide range of quantitative and qualitative assays were used to determine the suitability and evaluate the HEV RNA content of the candidate standards. Although the methods used by the study participants were all developed in-house, most assays consistently detected the 2 HEV strains. On the basis of data from the qualitative and quantitative assays, the candidate WHO IS was estimated to have a potency of 5.39 log_10_ units/mL. For practical purposes, the candidate IS was assigned a unitage of 250,000 International Units (IU)/mL; because the difference in the overall mean for the candidate Japanese national standard was negligible compared with the WHO preparation, the 2 materials were assigned the same value. In the case of the quantitative assays, laboratories reported values in HEV RNA copies/mL. The participating laboratories used plasmid DNA containing HEV sequences, synthetic oligonucleotides, and in vitro–transcribed HEV RNA to control for copy number. In some cases, laboratories used HEV-containing plasma that had been calibrated against in vitro–transcribed HEV RNA. One laboratory prepared a standard by using stool-derived virus, the titer of which was determined by endpoint dilution and analysis by Poisson distribution. No standard method or common quantitation standard material was used; this fact is reflected in the variation observed for the quantitative results (in the order of 2 log_10_), which were improved by expressing the results against sample 1 as a common standard. For qualitative assays, the variation in NAT-detectable units was >3 log_10_, and as with quantitative assays, expressing potencies relative to sample 1 improved the agreement among the different laboratories and methods.

Many of the laboratories participating in the study used a real time RT-PCR developed in 2006 (*34*) that was designed to detect the 4 main genotypes of HEV. However, a recent study in the United Kingdom found a polymorphism in the probe-binding site in several HEV-infected patients who initially had negative test results using this assay (*40*). A modification of the probe, increasing the melting temperature, restored detection of the polymorphic virus strains. We identified a further polymorphism in an HEV strain (GenBank accession no. JN995566) from a plasma donor (*18*), located in the probe-binding site of the same assay; use of the modified probe improved the amplification curve for this virus strain (S. Baylis and T. Gärtner, unpub. data). Genetic variation and its potential effects on HEV RNA detection highlight the importance of confirmatory tests of different design, rather than reliance on single methods.

The WHO IS will be valuable for development of secondary standards traceable to the IU, which will facilitate comparison of results between laboratories and determination of assay sensitivities and be helpful for validation purposes. We anticipate that the IS will find application in clinical laboratories, particularly in hepatitis reference laboratories that perform diagnosis and monitor HEV viral loads in chronically infected patients. The IS will also be helpful for research laboratories and blood and plasma centers that implement HEV NAT screening, regulatory agencies and organizations that are working to develop HEV vaccines, and manufacturers of HEV diagnostic kits.

The established WHO IS has been prepared by using a genotype 3a HEV strain. WHO has further endorsed a proposal by the PEI to prepare a genotype panel for HEV for NAT-based assays to continue standardization efforts for detection of this emerging infection. It is intended that the panel will contain representative strains of the 4 main genotypes of HEV that infect humans and notable subgenotypes. A new collaborative study will evaluate the IS against other genotypes and subgenotypes of HEV and investigate the commutability of the IS for standardization of assays for different genotypes of HEV. Laboratories that are able to provide high-titer HEV samples to aid in development of the proposed panel are requested to contact the authors.

In summary, WHO has established a genotype 3a HEV strain as the IS for HEV RNA (code number 6329/10), with an assigned a unitage of 250,000 IU/mL. The WHO IS for HEV RNA is available from PEI (www.pei.de).

Technical Appendix Assay protocols used by participant laboratories, mean estimates from quantitative assays (log_10_ copies/mL) and qualitative assays (log_10_ nucleic acid amplification technique detectable units/mL), and sample potency relative to sample 1 by quantitative and qualitative assays.
