# Supplementary material for: World Health Organization International Standard to Harmonize Assays for Detection of Hepatitis E Virus RNA
Source: Emerg Infect Dis. 2013 May;19(5):729–35. doi: 10.3201/eid1905.121845 (PMC3647515; doi:10.3201/eid1905.121845)
Supplement: Technical Appendix — Assay protocols used by participant laboratories, mean estimates from quantitative assays (log10 copies/mL) and qualitative assays (log10 nucleic acid amplification technique detectable units/mL), and sample potency relative to sample 1 by quantitative and qualitative assays. [file 12-1845-Techapp-s1.pdf]

Article DOI: http://dx.doi.org/10.3201/eid1905.121845

World Health Organization International Standard to Harmonize Assays for Detection of Hepatitis E Virus RNA

Technical Appendix

Technical Appendix Table 1. Assay protocols used by participating laboratories for evaluation of candidate hepatitis E virus standards*

Laboratory code	Assay type	Extraction method	NAT method	Assay target	Reference
1	Qualitative	QIAamp MinElute Virus Spin kit (QIAGEN)	Real-time RT-PCR (TaqMan)	ORF2/3	*1*
2	Qualitative/ quantitative	QIAamp Viral RNA Mini kit (QIAGEN)	Real-time RT-PCR (TaqMan)	ORF2	*2*
3	Qualitative/ quantitative	High Pure Viral Nucleic Acid kit (Roche)	Real-time RT-PCR (TaqMan)	ORF2/3	*1*
4	Qualitative	QIAamp Viral RNA Mini kit (QIAGEN)	Real-time RT-PCR (TaqMan)	ORF2/3	
5	Qualitative/ quantitative	QIAamp DNA Mini Blood kit (QIAGEN)	Real-time RT-PCR (TaqMan)	ORF2/3	
6	Quantitative	QIAamp Viral RNA Mini kit (QIAGEN)	Real-time RT-PCR (TaqMan)	ORF2/3	
7	Qualitative/ quantitative	QIAamp MinElute Virus Spin kit (QIAGEN)	Real-time RT-PCR (TaqMan)	ORF2/3	*3*
8	Quantitative	SMI-TEST EX-R&D (Medical Biological Laboratories Co., Ltd.)	Real-time RT-PCR (TaqMan)	ORF2/3	*4*
9	Qualitative/ quantitative	QIAamp Viral RNA Mini kit (QIAGEN)	Real-time RT-PCR (TaqMan)	ORF2/3	
10	Quantitative	COBAS AmpliPrep Total Nucleic Acid Isolation kit (Roche)	Real-time RT-PCR (TaqMan)	ORF2/3	*1*
11	Qualitative	COBAS AmpliScreen Multiprep Specimen Preparation and Control kit (Roche)	Conventional one step RT-PCR; analysis by agarose gel electrophoresis	ORF1	
12	Qualitative	QIAamp MinElute Virus Spin Kit (QIAGEN)	Real-time RT-PCR (TaqMan)	ORF2/3	*1*
13	Qualitative	QIAamp Viral RNA Mini kit (QIAGEN)	Real-time RT-PCR (TaqMan)	ORF2/3	*1*
14	Qualitative	Viral DNA/RNA Isolation kit (GenMag Biotechnology)	Nested RT-PCR; analysis by agarose gel electrophoresis	ORF2	
15	Qualitative/ quantitative	QIAamp Viral RNA Mini kit (QIAGEN)	Real-time RT-PCR (TaqMan)	ORF2/3	*1* (modified)
16a	Qualitative/ quantitative	MagNA Pure LC (Roche)	Real-time PCR (SYBR Green)	ORF2/3	*1* (modified)
16b	Qualitative	MagNA Pure LC (Roche)	Nested RT-PCR; analysis by agarose gel electrophoresis	ORF2	*5*
17	Qualitative/quantitative	QIAamp Virus BioRobot MDx kit (QIAGEN)	Real-time RT-PCR (TaqMan)	ORF2/3	*3*
18	Qualitative	MagNA Pure LC Total Nucleic Acid Isolation kit (Roche)	Real-time RT-PCR (TaqMan)	ORF2/3	*1*
19	Qualitative	easyMag (bioMérieux)	Real-time RT-PCR (TaqMan)	ORF2	
20	Quantitative	QIAamp Viral RNA Mini kit (QIAGEN)	Real-time RT-PCR (TaqMan)	ORF2/3	
21	Quantitative	BioRobot Universal (QIAGEN)	Real-time RT-PCR (TaqMan)	ORF2/3	*1*
22a	Qualitative	QIAamp RNA Mini kit (QIAGEN)	Nested RT-PCR; analysis by agarose gel electrophoresis	ORF2	*6* (modified)
22b	Qualitative	QIAamp RNA Mini kit	Real-time RT-PCR (TaqMan)	ORF2/3	*1* (modified)
23	Qualitative/ quantitative	QIAamp DNA Mini Blood kit (QIAGEN)	Real-time RT-PCR (TaqMan)	ORF2/3	*7*

*NAT, nucleic acid amplification technique; RT-PCR, reverse transcription PCR; ORF, open reading frame.

Technical Appendix Table 2. Mean estimates from quantitative assays (log_{10} copies/mL) determined for the candidate hepatitis E virus RNA standards*

Laboratory code	Sample			
	1	2	3	4
2	4.69	4.82	5.09	5.08
3	5.69	5.62	5.43	5.65
5	6.51	6.48	6.24	6.20
6	5.75	5.80	5.77	5.83
7	5.50	5.46	5.45	5.44
8	5.07	4.97	5.14	5.06
9	5.43	5.52	5.62	5.61
10	5.18	5.22	5.30	5.39
15	5.66	5.73	6.02	5.93
16a	5.59	5.62	5.64	5.51
17	5.40	5.34	5.35	5.41
20	5.70	5.65	5.74	5.65
21	5.25	5.23	5.25	5.23
23	6.54	6.53	6.31	6.41

Technical Appendix Table 3. Mean estimates from qualitative assays (log_{10} NAT detectable units/mL) determined for the candidate hepatitis E virus RNA standards*

Laboratory code	Sample			
	1	2	3	4
1	5.76	6.05	5.62	5.91
2	4.42	4.85	5.49	5.02
3	5.35	5.40	5.35	5.76
4	6.20	6.37	6.47	6.33
5	4.70	4.84	4.27	4.42
7	5.34	5.62	5.62	5.34
9	5.02	5.03	5.18	5.26
11		4.00	3.72	4.42
12	4.91	5.48	4.61	5.18
13	5.51	5.66	5.71	5.44
14	4.71	4.43	5.00	4.57
15	6.11	6.36	7.42	6.87
16a	5.32	5.17	5.17	5.17
16b	4.74	4.74	4.74	4.74
17	5.39	5.52	5.42	5.67
18	5.13	5.13	4.98	4.76
19	5.68	5.42	5.56	5.71
22a	5.21	4.92	4.91	5.44
22b	4.53	4.53	4.52	4.68
23	5.76	5.76	5.60	5.60

*NAT, nucleic acid amplification technique . Laboratory 11, sample 1, omitted due to 2 log_{10} higher cut-off.

Technical Appendix Table 4. Quantitative assay results for potency of samples 2, 3 and 4 relative to sample 1, the candidate WHO International Standard for HEV RNA for NAT-based assays*

Sample	Laboratory code	Relative potency (log_{10} copies/ml)	95% Confidence interval	
			Minimum	Maximum
2	2	5.54	5.29	5.78
	3	5.45	5.15	5.74
	5	5.39	5.15	5.63
	6	5.45	5.20	5.71
	7	5.38	5.28	5.47
	8	5.31	5.17	5.45
	9			
	10	5.47	5.34	5.59
	15	5.53	5.46	5.60
	16a	5.40	5.22	5.59
	17	5.36	5.29	5.43
	20	5.36	5.26	5.46
	21	5.39	5.35	5.44
	23	5.41	5.29	5.53
3	2	5.74	5.50	5.97
	3	5.36	5.07	5.65
	5	5.21	4.97	5.46
	6	5.48	5.21	5.75
	7	5.38	5.29	5.47
	8	5.55	5.41	5.69
	9			
	10	5.55	5.43	5.68
	15	5.83	5.76	5.90
	16a	5.55	5.36	5.73
	17	5.39	5.31	5.46
	20	5.52	5.42	5.62
	21	5.46	5.41	5.50
	23	5.20	5.09	5.32
4	2	5.90	5.66	6.15
	3	5.45	5.17	5.74
	5	5.17	4.93	5.42
	6	5.54	5.29	5.80
	7	5.37	5.28	5.46
	8	5.46	5.32	5.60
	9			
	10	5.63	5.50	5.76
	15	5.75	5.68	5.83
	16a	5.35	5.17	5.53
	17	5.44	5.37	5.52
	20	5.43	5.33	5.52
	21	5.44	5.39	5.48
	23	5.27	5.16	5.39

*It was not possible to estimate the relative potency for laboratory 9 since there were only two assay runs performed, each at a different dilution. WHO, World Health Organization; HEV, hepatitis E virus; NAT, nucleic acid amplification technique.

Technical Appendix Table 5. Qualitative assay results for potency of samples 2, 3 and 4 relative to sample 1, the candidate WHO International Standard for HEV RNA for NAT-based assays*

Sample	Laboratory code	Relative potency (log_{10} NAT detectable units/ml)	95% Confidence interval	
			Minimum	Maximum
2	1	5.68	5.10	6.27
	2	5.82	5.26	6.38
	3	5.44	4.81	6.08
	4	5.56	4.90	6.22
	5	5.53	5.09	5.97
	7	5.68	5.16	6.23
	9	5.40	5.15	5.66
	12	5.96	5.35	6.51
	13	5.54	5.14	5.91
	14	5.11	4.71	5.50
	15	5.65	4.90	6.40
	16a	5.24	4.85	5.64
	16b	5.39	4.77	6.01
	17	5.52	4.96	6.08
	18	5.39	4.88	5.90
	19	5.13	4.71	5.56
	22a	5.10	4.57	5.63
	22b	5.39	4.79	5.99
	23	5.39	4.74	6.04
3	1	5.25	4.67	5.81
	2	6.46	5.90	7.14
	3	5.39	4.76	6.02
	4	5.66	5.00	6.32
	5	4.96	4.53	5.39
	7	5.68	5.16	6.23
	9	5.55	5.30	5.80
	11	5.11	4.52	5.69
	12	5.09	4.51	5.64
	13	5.59	5.19	5.96
	14	5.67	5.27	6.08
	15	6.67	5.90	7.44
	16a	5.24	4.85	5.64
	16b	5.39	4.77	6.01
	17	5.43	4.87	5.98
	18	5.24	4.73	5.75
	19	5.28	4.85	5.70
	22a	5.10	4.56	5.63
	22b	5.38	4.78	5.97
	23	5.24	4.59	5.89
4	1	5.54	4.96	6.12
	2	5.99	5.43	6.55
	3	5.80	5.15	6.48
	4	5.52	4.86	6.18
	5	5.11	4.70	5.51
	7	5.39	4.87	5.92
	9	5.64	5.38	5.90
	11	5.81	5.23	6.40
	12	5.65	5.07	6.20
	13	5.32	4.93	5.71
	14	5.24	4.85	5.64
	15	6.13	5.39	6.88
	16a	5.24	4.85	5.64
	16b	5.39	4.77	6.01
	17	5.68	5.12	6.23
	18	5.02	4.51	5.52
	19	5.43	5.00	5.87
	22a	5.62	5.08	6.18
	22b	5.54	4.94	6.17
	23	5.24	4.59	5.89

*Relative potency from laboratory 11 was estimated relative to sample 2 (sample 1 had a cut-off 2 log_{10} dilutions higher). WHO, World Health Organization; HEV, hepatitis E virus; NAT, nucleic acid amplification technique.

References

1. Jothikumar N, Cromeans TL, Robertson BH, Meng XJ, Hill VR. A broadly reactive one-step real-time RT-PCR assay for rapid and sensitive detection of hepatitis E virus. J Virol Methods. 2006;131:65–71. PubMed http://dx.doi.org/10.1016/j.jviromet.2005.07.004

2. Adlhoch C, Kaiser M, Pauli G, Koch J, Meisel H. Indigenous hepatitis E virus infection of a plasma donor in Germany. Vox Sang. 2009;97:303–8. PubMed http://dx.doi.org/10.1111/j.1423-0410.2009.01211.x

3. Matsubayashi K, Kang JH, Sakata H, Takahashi K, Shindo M, Kato M, et al. A case of transfusion-transmitted hepatitis E caused by blood from a donor infected with hepatitis E virus via zoonotic food-borne route. Transfusion. 2008;48:1368–75. PubMed http://dx.doi.org/10.1111/j.1537-2995.2008.01722.x

4. Tanaka T, Takahashi M, Kusano E, Okamoto H. Development and evaluation of an efficient cell-culture system for hepatitis E virus. J Gen Virol. 2007;88:903–11.

5. Meng J, Dai X, Chang JC, Lopareva E, Pillot J, Fields HA, et al. Identification and characterization of the neutralization epitope(s) of the hepatitis E virus. Virology. 2001;288:203–11. PubMed http://dx.doi.org/10.1006/viro.2001.1093

6. Gyarmati P, Mohammed N, Norder H, Blomberg J, Belák S, Widén F. Universal detection of hepatitis E virus by two real-time PCR assays: TaqMan and Primer-Probe Energy Transfer. J Virol Methods. 2007;146:226–35. PubMed http://dx.doi.org/10.1016/j.jviromet.2007.07.014

7. Wenzel JJ, Preiss J, Schemmerer M, Huber B, Plentz A, Jilg W. Detection of hepatitis E virus (HEV) from porcine livers in Southeastern Germany and high sequence homology to human HEV isolates. J Clin Virol. 2011;52:50–4. PubMed http://dx.doi.org/10.1016/j.jcv.2011.06.006
